# Asking about the last four drinking occasions on a tablet computer as a way to record alcohol consumption in Aboriginal and Torres Strait Islander Australians: a validation

**DOI:** 10.1186/s13722-019-0148-2

**Published:** 2019-05-01

**Authors:** K. S. Kylie Lee, James H. Conigrave, Sarah Callinan, Scott Wilson, Robin Room, Jimmy Perry, Tim Slade, Tanya N. Chikritzhs, Noel Hayman, Teagan Weatherall, Geoffrey Leggat, Dennis Gray, Katherine M. Conigrave

**Affiliations:** 1 NHMRC Centre of Research Excellence in Indigenous Health and Alcohol, Discipline of Addiction Medicine, Faculty of Medicine and Health, The University of Sydney, King George V Building, 83-117 Missenden Road, Camperdown, NSW 2050 Australia; 2 Centre for Alcohol Policy Research, La Trobe University, Melbourne, VIC Australia; 3 Aboriginal Drug and Alcohol Council (ADAC) South Australia, Adelaide, Australia; 4 The Matilda Centre for Research in Mental Health and Substance Use, Faculty of Medicine and Health, The University of Sydney, Sydney, NSW Australia; 5 Health Sciences, National Drug Research Institute, Curtin University, Perth, WA Australia; 6 Southern Queensland Centre of Excellence in Aboriginal and Torres Strait Islander Primary Health Care, Inala, QLD Australia; 7 School of Medicine, Griffith University, Brisbane, QLD Australia; 8 School of Medicine, University of Queensland, Brisbane, QLD Australia; 9 Drug Health Services, Sydney Local Health District, Royal Prince Alfred Hospital, Sydney, NSW Australia

**Keywords:** Aboriginal, Indigenous, Alcohol, Consumption, Measurement, Survey

## Abstract

**Background:**

Alcohol consumption among Indigenous Australians can be irregular, depending on social and geographic context. The Finnish method uses the last four drinking occasions to estimate drinking quantity and pattern. The Grog Survey App is an interactive and visual tablet computer application which uses touch-screen technology to deliver questions on drinking.

**Methods:**

Alcohol consumption recorded on the Grog Survey App using the last four occasions (Finnish) method was compared with a clinical interview conducted by an Indigenous Australian health professional. To assess convergent validity, Spearman’s ranked correlations between consumption estimates from the App and from interview were calculated. Sensitivity and specificity analyses were used to compare how well the App and clinical interview agreed when classifying drinkers’ risk. To assess criterion validity, average grams alcohol per day as estimated by the App (and by interview) were compared against presence of self-reported withdrawal tremors (from App or interview). Test–retest reliability was assessed by correlations between measures of alcohol consumption recorded on two occasions.

**Results:**

The App recorded higher numbers of standard drinks consumed per drinking occasion than the interview. There was reasonable agreement between the App and interview across common reference periods (sensitivity 92.7%, specificity 69.8%, short-term risk; sensitivity 70.7%, specificity 68.8%, long-term risk). Average consumption recorded by the App was as good or better predictor of withdrawal tremors than consumption as estimated by interview.

**Conclusions:**

The Finnish method, as delivered by the App, offers an innovative way to collect survey data on alcohol in a population with an intermittent drinking pattern.

## Background

Alcohol is consistently reported as a key concern for Aboriginal and Torres Strait Islander (Indigenous) peoples in Australia [1, 2] and for many indigenous populations worldwide [3]. Reliable population estimates of alcohol use among Indigenous peoples are needed to inform efforts to prevent and treat risky drinking or alcohol use disorders. However, estimates from the largest (and often quoted) national survey in Australia have been reported to underestimate alcohol consumption by over 200% for Indigenous males and 700% for females [4]. Deficits in this and other household surveys [4–6] may have contributed to chronic under-funding of alcohol prevention and treatment services.

A range of survey items have been used to assess alcohol consumption in general populations. Most include an assessment of the overall frequency of drinking and the usual quantity consumed on each occasion (quantity-frequency), or alternatively, how often certain amounts of alcohol are consumed on a weekly or monthly basis (graduated-frequency) [7]. Both methods pose challenges with a population such as Indigenous Australians, and other culturally and linguistically diverse peoples, where drinking is often episodic and irregular [8] and where there is not necessarily a ‘usual’ drinking pattern. The timing of drinking may be influenced by unpredictable circumstances such as funerals. ‘Timeline Followback’ is a detailed retrospective diary, where the participant is asked to describe the circumstances and level of consumption of each drinking occasion during a specific timeframe [9]. However this can be relatively time consuming to deliver [10]. An alternative, the ‘Finnish’ method, asks about alcohol consumption on the last four drinking occasions [11]. Both Timeline Followback and Finnish methods can be delivered in a conversational approach that may be suited to the storytelling traditions of Indigenous peoples.

Most of these approaches to assessing drinking ask participants to describe their consumption in ‘standard’ drinks [6], requiring mental arithmetic for conversions. Even in general populations, some authors suggest that self-reporting standard drinks (rather than specific containers and types of alcohol) may lead to underestimates of consumption [12]. Indigenous Australians may use a wide range of drinking containers, each holding from between 1.5 and 20 standard drinks, making the conversion more difficult. Also, in settings where sharing of alcohol is common, the challenges of estimating quantity consumed are increased [6].

There is a lack of validated tools to assess alcohol consumption in Australia’s Indigenous peoples, or in similarly colonised peoples (e.g. Canadian First Nations or New Zealand Māori). It is unclear whether tools validated for non-Indigenous settings are suitable in these contexts [13, 14]. A small number of tools have been developed specifically to collect alcohol consumption data in Indigenous Australians but validation data are minimal. For example in remote Western Australia (WA), the Questionnaire for Alcohol Research in the Kimberley (QARK) [15] distinguishes between intermittent drinking (i.e. around regular occasions, like payday), and episodic drinking (i.e. irregular or sporadic occasions). It drew on Timeline Followback elements to ask about drinking contexts. While the validity of QARK was checked during survey development, including by comparison with alcohol sales figures, validation data were not published. In another survey on alcohol (and other drug use) 10% of Indigenous people in the Northern Territory (NT Australia; non-urban areas) were interviewed [16]. The alcohol questions were loosely based on a quantity-frequency measure with an additional one-week retrospective diary. Questions were tailored to the population (e.g. providing images of non-standard drinking containers; allowing for sharing of drinks; and distinction between pay-/pension-week versus non-pay/non-pension-week). However, no formal validation study was conducted.

A number of shorter screens for risky drinking have been used among Indigenous Australians, including the first three (consumption) questions of the Alcohol Use Disorders Identification Test (AUDIT-C) [17, 18]. However, data on how consumption assessed in this way compared with estimates from other methods or ‘gold standards’ are limited. In regional New South Wales (NSW, Australia), one study showed that a modified version of AUDIT-3 captured a longer period of drinking than did a 7-day retrospective diary, although AUDIT-3’s response categories provide only broad information on drinking. Furthermore, AUDIT’s frequency response categories assume drinking regularity [19]. The 7-day retrospective diary method (as delivered on a touchscreen computer in an Aboriginal community controlled health service) missed nearly a third (31%) of current drinkers as they did not consume any alcohol in the last week [20].

In any population, collecting reliable self-report data on alcohol use is complex given the potential sensitivity of this topic. These concerns can be greater among Indigenous populations because of experience of racism, fear of consequences of admitting to heavy drinking, and shame over harms from drinking [21]. For all these reasons, it is important to ensure methods to collect drinking data ensure privacy and are appropriate in a cross-cultural context [13]. In studies of sensitive topics, touch-screen tablet devices may increase confidence in confidentiality and anonymity [21–23]. In addition, programming can cater for lack of comfort with written language or numeracy in populations which are educationally disadvantaged or do not speak the majority language. Visual images can allow participants to estimate container sizes for alcohol and sharing of drinks [21]. Tablet devices can also reduce missing data [24] and potentially streamline the survey experience for both participants and research assistants. In addition, pre-recorded translation can remove the need for and expense of translation at the point of survey administration, and can help to standardise translation and maximise respondent privacy [22].

The Grog Survey App [the App] was developed in response to the need for an easy-to-use tool to help Indigenous Australians report on their drinking in a household survey environment [21]. “Grog” is a colloquial name for alcohol. The App presents an adaptation of the Finnish method to ask participants to describe their drinking on the last four occasions in the past 12 months [11]. The current study compares alcohol consumption as estimated by the Finnish method and recorded on the App with a clinical interview conducted by an Aboriginal health professional. As there are no validated instruments to measure alcohol consumption in Indigenous Australians, clinical interview by a health professional with understanding of local culture and context was chosen as a recognised [25–27] and culturally appropriate method.

## Methods

Study methods were designed by investigators in consultation with the Aboriginal Drug and Alcohol Council of South Australia; the Aboriginal Drug and Alcohol Network, representing Aboriginal alcohol and other drug workers in NSW; and the Aboriginal Health Council of South Australia (AHCSA), the peak body for ACCHSs in South Australia (SA). Ethical approval was obtained from ACHSA and from the Metro South Health Human Research Ethics Committee in Queensland.

### Recruitment

To assess the validity of the Finnish method as delivered by the App with a range of drinkers, stratified sampling was used. We aimed to recruit: 20 non-drinkers, 40 non-dependent drinkers and 40 dependent drinkers in each of two states by word-of-mouth in each service. Most of the analyses involved in validating and shortening the scale used in the pilot study require little statistical power. For instance, for the reliability analysis, in order to have sufficient power (80%) to identify a correlation of 0.4 where *r* = 0.8, and ɑ = .05 a sample size of 46 is required (calculated using the ‘pwr’ package in R). Greater sizes were sought to try to allow analysis of differences between urban and remote sites in sampling, and because of anticipated challenges in ensuring complete data collection. In urban Queensland (Qld), recruitment was based in an Indigenous primary health care service and surrounding community. In South Australia (SA), recruitment centred on a regional ACCHS and a remote Aboriginal community-controlled drug and alcohol day centre (a drop-in service). Individuals were eligible for inclusion if they self-identified as being Aboriginal or Torres Strait Islander and were 16+ years. Exclusion criteria included obvious intoxication. Participants were reimbursed for their time and travel expenses with a store voucher.

Each participant also took part in a semi-structured clinical interview, typically within 2–7 days of completing the App. We set out to have half the participants complete the App before the interview, and half afterwards. To assess the test–retest reliability of App responses, participants were asked to complete the App twice within 2–7 days.

### Data collection and instruments

#### Grog Survey App

The development of the App and selection of its survey items have been described elsewhere [21]. Broadly, the App presented questions on demographics, alcohol consumption (10 items), alcohol dependence (3 items based on ICD-11 [28]), harms to self or others, treatment access and participants’ feedback on using the App.

The App ‘reads out’ the questions in English or in Pitjantjatjara (a language spoken in a region of NT, SA and WA). The App was designed to take no longer than 20 min to complete. Aboriginal field research assistants handed the tablet computer to participants, with brief guidance, then stood to one side, in case there were any challenges. Individuals with no prior computer contact were able to use the App without assistance [29]. The App is designed to work ‘offline’ (without access to the internet) and data are synchronised at the end of each working day to a secure encrypted server at the University of Sydney.

- Alcohol consumption

Using an adaptation of the Finnish method of assessing drinking, the App asks respondents to show the date of their four most recent drinking occasions within the last twelve months. Participants are then asked how much alcohol they consumed on each of these occasions. Participants select pictures of the type of alcohol, the container they drank it out of, and how full the container was with alcohol [21]. The App also allowed the participant to describe the alcohol consumption of their drinking group, if that was easier for them, and to then show their share.

- Alcohol withdrawal tremors (‘grog shakes’)

Participants were asked: “Some people’s hands shake when they stop drinking or before their first drink of the day. How often does this happen to you?” Responses were indicated on a five-point Likert scale ranging from ‘never’ to ‘most days or every day’.

Data collection for the survey App was facilitated by five Aboriginal research assistants (1 male and 2 female, SA; 2 female, Qld) who were either Aboriginal health workers [3] or Aboriginal health professionals working in drug and alcohol [2]. One day of face-to-face training was provided on how to use the App and on study methods. These research assistants introduced each participant to the App then sat a short distance away to ensure privacy, ready to respond if questions arose.

#### Clinical interview

The clinical interview was conducted by two female Aboriginal health professionals (one in each state), each with knowledge of local culture, context and language. In keeping with past research with Aboriginal respondents [30], this was considered the most suitable reference standard. It was not considered appropriate to have a non-Indigenous addiction psychiatrist or psychologist, as the gulf in cultural understanding and trust can interfere with assessment quality, particularly in remote regions, but even in urban settings [25]. A semi-structured framework was used by the health professional to record notes on their findings (available from the authors on request). This framework was derived from one used by a respected Aboriginal alcohol and drug worker in regional NSW. It was adapted with the help of the two Aboriginal health professionals, in order to better fit the needs of the recruitment sites. The two Aboriginal health professionals agreed on the goal of a conversational clinical interview of 30 min or less.

Consistent with local clinical practice, the interviewer assessment of drinking focused on the past 14 days. Notes on drinking quantity were recorded on a 2-week calendar showing the days of the week (from Monday to Sunday in each row) so that the client could look along with the interviewer. The approach used was similar to the Timeline Followback [9]. If no drinking, or atypical drinking had taken place in the previous 1–2 weeks, the interviewer asked about an additional 2 weeks (i.e. 3–4 weeks in total).

On each drinking day, the interviewer recorded ‘longhand’ what the participant drank, for example, “two longnecks of West End”, which is the local term for 2 × 750 mL bottles of a brand of full-strength beer. To maximise participant engagement and to keep the interview short, interviewers did not convert responses to Australian standard drinks (10 g ethanol). This was later done by a research assistant (TW).

Interviewers also assessed dependence, by asking current drinkers if they experienced tremors when they stop drinking or cut down. Clarification on the interviewers’ clinical notes was sought by one author (KC) if needed. Data entry was conducted by a research assistant.

### Analysis

Standard drinks were calculated by the App itself. All other analyses were performed in the R statistical programming language [31]. As there was a lack of a clear gold standard in use identified for Indigenous samples, validity was determined by triangulating the Finnish method with multiple outcomes. The primary outcomes used to compare the Grog App and clinical interview, was the classification of drinking risk, as defined by current Australian guidelines [32]. We also looked at the Spearman correlations of estimates of consumption from each measure with each other, and to the frequency of withdrawal tremors. Steiger’s z-test [33] was used to test for significant differences between the correlations of consumption data and the presence of withdrawal tremors, between the App and clinical interview.

The mean number of standard drinks that participants consumed on drinking occasions was calculated by taking the average of the number of standard drinks consumed on the most recent four drinking occasions. The last four occasions (Finnish) method of assessing consumption does not use a fixed reference period. Non-drinking days are recorded between the interview and most recent occasion. However, necessarily, the reference period ends with the fourth most recent drinking occasion. On average, this results in over-estimates of drinking frequency. To reduce this bias, for each individual, we extended the reference time period by adding half of the average gap between their drinking occasions to their reference period. To calculate the frequency of drinking occasions, the total number of recorded occasions was then divided by the total reference period (in days). To calculate average daily consumption, we multiplied the average quantity each individual consumed per occasion, by their frequency of drinking occasions.

Following data cleaning, estimates of alcohol consumption by the last four occasions method were compared to estimates from the clinical interview. We examined the correlation between the two estimates of both drinking intensity (drinks per drinking occasion) and average daily consumption for each person.

Sensitivity and specificity were calculated to compare the extent to which the App agreed with the clinical interview when classifying drinkers’ risk (as defined by the National Health and Medical Research Council [32]). The clinical interview was used as the reference standard. To assess convergent validity, we compared alcohol consumption estimated by the App, against the presence of withdrawal tremors (recorded on the App or in the interview). We also compared consumption measured by clinical interview against withdrawal tremors. Finally, test–retest reliability was assessed by correlating consumption across the two occasions when participants used the App.

#### Classification of drinking risk

Short-term risk from drinking was defined as consumption of more than four drinks on any occasion [32]. Long-term risk from drinking was defined as average consumption of more than two drinks per day [32]. Both these criteria are in keeping with Australian guidelines to reduce the risk from drinking [32]. In addition, a short-term high risk threshold of more than 10 drinks per day was examined based on the higher odds of motor vehicle accidents at 11+ drinks per day in a meta-analysis [34]. Long-term high risk was defined by average daily consumption of more than five standard drinks was examined. This cutoff was based on a meta-analysis showing increased relative risk of cancers at that level [35].

#### Reference period matching

Comparisons of App and interview consumption initially considered all recorded drinking data. However, because the clinical interview focused on the last 7–29 days while the App collected data on four occasions which could be spread across the year, comparisons were then repeated using only those days which were examined by both the App and interview. To account for differences in participants’ recall of when these occasions occurred, a buffer of 3 days was included. For example, if a date logged in the App occurred within 3 days of a date in the corresponding clinical interview (whether consumption was zero or higher), it was included in the analyses. The median number of matched days was 23.5. All participants had one or more matched days.

## Results

### Overview

#### Data cleaning and exclusions

A total of 238 participants completed both the App and clinical interview. Of these, 32 cases were excluded because the hand-recorded information required to match the individuals’ interview and App responses was incomplete or incorrect (gender, age or interview location). Most of these cases were from a remote location where external events disrupted the pen-and-paper participant registration. After exclusions the final sample size was 206 participants.

#### Handling of outliers

Twelve participants reported drinking more than 100 Australian standard drinks on a single occasion on the App. These quantities seem unlikely (based on clinical experience [KC], and personal communication with a medical director of a residential detoxification unit). However, as we are using non-parametric analytic methods the influence of outliers is minimal. Accordingly, these cases were included in the analyses initially, then analyses repeated with them excluded.

#### Sample characteristics

Just over half (51.9%) of the sample were male. Ages ranged from 18 to 78 years (mean = 39.3; SD = 14.6). Almost half (47.6%) of the sample were from urban areas; 16.5% from regional, and more than a third (35.6%) from remote areas (Table 1). More than four in ten (42.2%, n = 87) participants completed the App first, while nearly six in ten (57.3%, n = 118) completed the interview first. In one case it was not clear which was completed first (time not recorded on the interview notes). Gender, drinking status, and age did not vary based on whether subjects completed the interview or App first (two Chi squares and a spearman correlation respectively; all *p* > .9; calculations used app data, these relationships were also non-significant using interview data).

**Table 1:** Characteristics of Aboriginal or Torres Strait Islander participants who completed the Grog Survey App (n = 206)

Variable	n	Percent
Sex		
Male	107	51.9
Female	99	48.1
Age (years)		
16–19	8	3.9
20–39	106	51.5
40–59	71	34.5
60+	21	10.2
Remoteness		
Regional	34	28.2
Remote	74	47.1
Urban	94	24.8
Highest completed school year		
Year 9 or below	58	28.2
Year 10 or Year 11	97	47.1
Year 12	51	24.8
Employment status		
Employed	77	37.4
Unemployed	129	62.6
Total	206	100.0

### Drinking status

The App and interview agreed on participants’ drinking status in 94.7% of individuals. In total, 156 participants (75.7%) were identified as having consumed alcohol in the last 12 months by both the App and interview. The App identified more participants as drinkers than the interview—80.6% (n = 166) and 76.2% (n = 157) respectively. Interviewers recorded at least 14 days of drinking data for 86.5% of drinking participants. For the majority of drinkers (75.4%), the Grog App covered a reference period equal to, or longer than the clinical interview.

### Consumption level

The App tended to record a higher median number of standard drinks per drinking occasion than the clinical interview (median = 17.0, IQR = [10.5, 27.9]; median = 15.4, IQR = [9.6, 23.2], respectively, matched reference periods). The greatest number of standard drinks consumed on a single occasion for each participant also tended to be higher for the App than for the interview (*median* = 14.8, *IQR* = [4.1, 28.4]; *median* = 14.0, *IQR* = [1.8, 23.2]; respectively, matched reference periods). On the App, males and female drinkers recorded a similar median number of standard drinks per occasion (males 17.0, *IQR *= [11.8, 28.4]; females 16.5. *IQR* = [7.7, 26.0]; matched reference periods). In the interview, male drinkers reported a higher median number of standard drinks per occasion than female drinkers (males 16.6, *IQR* = [11.5, 26.7], females 12.8, *IQR* = [8.0, 22.1]; matched reference periods).

Across all data, drinkers on the App recorded a median of four drinking occasions (*IQR* = [4.0, 4.0]) per participant compared with one (*IQR* = [1.0, 3.0]) for the interview. In keeping with this, nearly one in five (18.6%, n = 29) of those who reported consuming any alcohol in the last 12 months on the App did not report any drinking occasions during the four-week retrospective diary. All of these participants recorded at least one drinking occasion on the App.

Because most drinkers were not daily drinkers, despite high drinks per drinking day, average daily consumption was low. The recorded average consumption was slightly higher on the App than on clinical interview (App *median *=1.5, *IQR* = [0.3, 3.8]; *median* of 1.1, *IQR *= [0.2, 2.1]; respectively; matched data).

### Proportion of participants classified as risky drinkers

As the App recorded a higher quantity per drinking occasion, and a higher number of occasions, it tended to identify slightly more participants as being at risk, across all risk classifications, even when the time frames examined were matched (Table 2).

**Table 2:** Percentage of drinkers classified at each level of risk

	All data		Matched time periods	
	App	Interview	App	Interview
Short-term risk	95.5	76.3	75.2	71.9
Long-term risk	48.1	28.2	41.8	26.8
High-risk: short-term	81.4	64.7	62.7	62.1
High-risk: long-term	22.4	8.3	21.6	10.5

Only participants who completed both App and interview were included, who were identified as drinkers in both App and interview (n = 156)

During the matched data periods, the App found 76.7% of drinking males and 69.7% of females met one or more criteria for being at risk, compared to the interview which identified 74.4% of males and 65.2% of females as at risk.

### Agreement between consumption data

The average daily consumption recorded for individuals by the App and by interview was moderately correlated (r = .52). Correlations were greater when only data from matched time periods were used (r = .62) (Table 3).

**Table 3:** Correlations in reported amount consumed between App and interview

Drinker status	All data	Matched time periods
All participants	.68*	.72*
Drinkers	.52*	.62*

*p < 0.01; Correlations are Spearman rho (r_s_). n = 206, and 156, all participants and drinkers, respectively

The App appeared to be highly sensitive in detecting at risk drinkers, as identified by the clinical interview, across all risk categories. The App detected 37 more risky drinkers than the interview if time periods were not matched. However, when only common reference periods were used the App identified only 10 more people at risk. This corresponded to an improvement in both sensitivity and specificity. Across all risk categories, when all data from drinkers was used, the sensitivity was 97.5% and the specificity was 10.8%; (95% CI [92.8, 99.5] and [3.03, 25.4], respectively). When common reference periods were used, across all risk categories, the sensitivity was 92.7% and the specificity was 69.7% (95% CI [86.2, 96.8], and [53.9, 82.8] respectively). The sensitivity and specificity for each risk category is presented in Table 4.

**Table 4:** Sensitivity and specificity of the App in detection of risky drinking, where the clinical interview is used as the reference test

Risk	All data		Matched time periods	
	Sens.	Spec.	Sens.	Spec.
Short-term risk	0.97 [0.93, 0.99]	0.11 [0.03, 0.25]	0.93 [0.86, 0.97]	0.70 [0.54, 0.83]
Long-term risk	0.70 [0.55, 0.83]	0.61 [0.51, 0.70]	0.71 [0.54, 0.84]	0.69 [0.59, 0.77]
High-risk: short-term	0.93 [0.86, 0.97]	0.40 [0.27, 0.54]	0.88 [0.80, 0.94]	0.79 [0.67, 0.89]
High-risk: long-term	0.69 [0.39, 0.91]	0.82 [0.75, 0.88]	0.81 [0.54, 0.96]	0.85 [0.78, 0.91]

Sens = Sensitivity; Spec = Specificity; drinking subjects only; n = 156; 95% confidence intervals are presented within square brackets

### Correlation of average daily alcohol consumption and withdrawal tremors

To assess criterion validity of the App’s estimate of alcohol consumption, we compared average daily consumption measured by the App (and by the interview) with the presence of withdrawal tremors, as recorded on either the App or interview. Participants were more likely to report the presence of tremors when using the App than when responding to the interview (17.0% and 7.2% respectively; r_s_ = 0.48).

Average consumption recorded by the App was as good or better a predictor of withdrawal tremors than was consumption recorded by the interview (Table 5). This was consistent regardless of whether tremors were recorded by the App or interview (*p* = 0.02 and 0.44 respectively; Steiger’s z-test).

**Table 5:** The relationship between alcohol withdrawal tremors and average alcohol consumption per day for participants at long-term risk

	Consumption recorded by App	Consumption recorded by interview
Tremors reported on App	0.51*	0.29*
Tremors reported on interview	0.40*	0.32*

*p < .05; correlations are Spearman rho; n = 88. This analysis only considers matched time periods as symptoms of dependence are sensitive to whether heavy drinking was recent or not

### Grog Survey App test–retest

In total, 194 participants completed the App twice, with a median of three days (*IQR* = [2.0, 6.0]) between administrations (range 1–51 days). Almost eight out of 10 (78.4%, n = 152) participants completed the App twice within a week. Average consumption was well correlated across the two App administrations (r_s_ = .81, n = 181, all participants; r_s_ = .81, n = 147, drinkers only).

## Discussion

To our knowledge, this is the first study to formally report on testing the validity of an instrument designed to assess alcohol consumption in detail in Aboriginal and Torres Strait Islander Australians, or perhaps in Indigenous populations worldwide. The Finnish method of assessing consumption appears well suited to drinking which may be irregular and tied to external or social events. The App offers an innovative and interactive way to deliver these alcohol consumption questions. It appears to offer considerable potential to collect household survey data on drinking in a population where existing approaches have been found unsuitable [5, 36].

### Finnish method used by the App versus retrospective diary in the clinical interview

The Finnish method as administered on the App had high sensitivity for short-term risk (0.93, 0.88; 95% CI [0.86, 0.97], and [0.80, 0.94], respectively; NHMRC short-term risk and high-risk short-term; matched time periods). When time periods were matched, the App also had good specificity compared with the clinical interview for long-term risk (0.69, 0.85; 95% CI [0.59, 0.77], and [0.78, 0.91], respectively; for NHMRC long-term risk and high-risk long-term). Low specificity for short-term risk (0.11, 0.40 for risk and high-risk respectively; 95% CI [0.03, 0.25] and [0.27, 0.54]) is likely to be due to the longer reference period (up to 12 months) in those who completed the App compared with those who completed a clinical interview (2–4 weeks). Consumption as estimated by the App appears well correlated with alcohol withdrawal tremors. This suggests both internal consistency (in the case of within-App comparison), and criterion validity (in the case of comparison with tremor as reported in the clinical interview). Test re-test reliability was also good, particularly given that consumption may vary from day-to-day.

The App recorded higher median consumption per drinking occasion than the retrospective diary did in the clinical interview, even when examining matched time periods. There are a number of reasons that suggest that the higher prevalence of risky drinking estimated by the App may be more accurate than the clinical interview. Firstly, this is consistent with the high prevalence of alcohol-related harms observed in Indigenous Australians [2]. Secondly, past research shows that the manual conversion of beverages consumed and drinking containers into standard drinks often results in an under-reporting of quantity [37]. The App uses a visual and interactive system to record drinking quantity and computes the standard drink equivalent. Slightly more participants undertook the interview first which could have inflated performance of App or explain higher consumption reported on the App (i.e. by memory prompting).

The longer reporting period offered by the last four occasions method, as delivered on the App, compared with interview allows greater ability to detect infrequent, irregular, episodic drinking that is still high quantity. This pattern is common in this population [38]. A standard quantity-frequency method (e.g. asking how many standard drinks a person usually consumes; or how often they drink above a risk threshold) may miss high-risk episodic drinking occasions. It may also be difficult for irregular drinkers to answer questions on frequency of drinking. For instance, a drinker who consumes alcohol for 12 days in a row then nothing for 90 days may have difficulty selecting from survey response options such as “weekly” or “monthly”.

### Comparison with other tools to measure alcohol consumption

There are similarities between the Finnish method as delivered on the App and paper-and-pen survey tools previously used to record detailed alcohol consumption in Indigenous Australians in remote WA [15] and non-urban NT [16]. These older surveys both made no assumptions about ‘regular’ drinking patterns and provided options to enquire about episodic drinking (e.g. during pay/pension weeks). Both also enquired about sharing of drinks and asked about drinking context and incorporated a visual approach (e.g. pictures of alcohol types or common containers). Accordingly, they did not assume comfort with mental arithmetic or literacy.

The App also has similarities with an approach developed in Thailand to measure alcohol consumption that varies with cultural or other holiday periods in Thailand—the context-specific quantity-frequency method [39].

### Tablet computer as the delivery tool

Computer administration means that separate data entry of responses is not required, which reduces opportunity for human error. Computer administration also takes away the need for participants or interviewers to follow complicated survey instructions (e.g. ‘If no, skip to question 12’). Audio recordings enable standardisation of verbal presentation of questions.

### Limitations

Just a small proportion (an estimated 10%) of individuals approached declined to take part in the study. However, reasons for refusal were not systematically collected.

We chose to compare the App to a clinical interview. However, there is no perfect ‘gold standard’ against which the App can be compared. We cannot assume a mainstream gold standard, such as CIDI-SAM [40] will be valid in this context [13, 30]. Clinical interview by an Indigenous health professional is recognised as a valid reference standard for research in Indigenous settings [25, 30]. It allowed communication which avoided major cultural and language gulfs. This same gold standard was used in the development of the Indigenous Risk Impact Screen (IRIS), a tool to measure alcohol and other drug and mental disorders risk [30]. As in our study, in Schlesinger’s [30] the clinical interviews were conducted by experienced Indigenous (generalist) health professionals rather than alcohol and other drug workers. Specialised Indigenous alcohol and other drug workers are not available in all communities around Australia.

The clinical assessment against which the App was compared included a 2–4 week retrospective diary. Retrospective diaries are commonly used to assess Aboriginal and Torres Strait Islander drinking patterns in clinical practice and in research. In research settings shorter reference periods are often used to shorten interview time, or to avoid over-reliance on memory. In this sample, the Grog App reported that several participants only drank a few times a year (albeit in large quantities). As a result, the 2–4 week retrospective diary recorded during the clinical interview, would not have adequately captured the drinking patterns of those who had not regularly consumed alcohol over that time period.

To adjust for this limitation, our sub-analyses were restricted to data from the App which was recorded in the same reference periods as recorded in the clinical interview. While this resulted in data-loss, as expected, it greatly improved sensitivity and specificity of the Grog App compared to the retrospective diary. This finding is relevant to researchers and clinicians, indicating that in samples with irregular drinking, detection of risky drinking may become less reliable with shorter reference periods. In future research it will be useful to compare the findings of the Grog App to instruments which may be better able to capture irregular drinking patterns. One potential example is AUDIT-C, though as discussed earlier, most response categories on this instrument assume some regularity of drinking.

Despite using stratified sampling, the research assistants were only able to recruit small numbers of drinkers who were drinking within recommended limits. It would be good to repeat this validation with a sample with a greater range of drinking patterns. For example, a sample with higher socio-economic stratum, may have a greater range of drinking patterns [41].

Fourteen individuals reported what we considered unrealistically high amounts per drinking occasion (100 + standard drinks) on the App. On clinical interview, all of these individuals were classified at short-term risk (median of 22.5 standard drinks per drinking occasion) and 79% at long-term risk from drinking. While it is not clear why these individuals reported drinking such high amounts on the App, repeating the analysis with their exclusion did not greatly change results.

Future research could use additional sources of validation as set out in the LEAD paradigm (Longitudinal, Expert and All Data), such as longitudinal data on which drinkers experience harms over the coming months or years [42]. Use of sales data for validation is difficult, as few communities with high proportions of Indigenous individuals in Australia currently have unrestricted access to local alcohol, so supply may be illicit.

### Implications for policy, practice and further research

This survey tool is likely to be valuable not just for Indigenous Australians, but in other contexts, such as developing countries, where alcohol is consumed in non-standard containers or episodically. Even for general populations, the visual interface provided by the App greatly simplifies reporting, regardless of comfort with writing or numbers. The tablet-computer platform is likely to save errors in data recording and data entry [24]. This is particularly important in remote communities or in the developing world, where the interview environment is often not a controlled one.

There has been considerable interest from Aboriginal and Torres Strait Islander primary healthcare services to use the App as a screening tool while clients wait to be seen by their health professional. Next steps are to refine the App based on suggestions received during the testing phase. Then the practical feasibility of the App as a survey tool will be examined. Further research on shortened versions to use as clinical screening tools will also occur. The survey App will be made available as a private iPad download from the ‘App Store’ initially, to ensure ongoing quality control. Features in this refined version will include options for individuals to generate a summary report on their answers (which could then be shared with their health professional, with that individual’s consent). It will also include a feature for communities to collect their own Grog App data, and to generate a community-level summary report on drinking in their community.

## Conclusion

The Finnish method of assessing drinking as delivered on the Grog Survey App is the first approach to be formally tested for collecting a detailed assessment of alcohol consumption in Indigenous Australians, and possibly in Indigenous peoples worldwide. The last four occasions method appears to better cater for episodic and irregular drinking than a retrospective diary (as is often used in clinical interview). The visual and interactive interface of the App is likely to assist with accuracy and comfort with reporting drinking, regardless of literacy or numeracy. This household survey tool is likely to be of interest to researchers, communities, health services and policy-makers seeking to assess alcohol consumption in vulnerable and marginalised populations worldwide.
